# Thermal Insulating Rigid Polyurethane Foams with Bio-Polyol from Rapeseed Oil Modified by Phosphorus Additive and Reactive Flame Retardants

**DOI:** 10.3390/ijms232012386

**Published:** 2022-10-16

**Authors:** Marcin Zemła, Aleksander Prociak, Sławomir Michałowski, Ugis Cabulis, Mikelis Kirpluks, Kirils Simakovs

**Affiliations:** 1 Department of Chemistry and Technology of Polymers, Cracow University of Technology, Warszawska 24, 31-155 Cracow, Poland; 2 Latvian State Institute of Wood Chemistry, Dzerbenes 27, LV-1006 Riga, Latvia

**Keywords:** rigid polyurethane foams, bio-polyol, halogen-free, flammability, thermal conductivity, flame retardant

## Abstract

In this article, rigid polyurethane foams obtained with the addition of a bio-polyol from rapeseed oil, were modified with the dimethyl propane phosphonate as additive flame retardant and two reactive flame retardants diethyl (hydroxymethyl)phosphonate and diethyl bis-(2-hydroxyethyl)-aminomethylphosphonate. The influence of used flame retardants on the foaming process and characteristic processing times of tested polyurethane systems were determined. The obtained foams were tested in terms of cell structure, physical and mechanical properties, as well as flammability. Modified foams had worse mechanical and thermal insulation properties, caused by lower cellular density and higher anisotropy coefficient in the cross-section parallel to the foam rise direction, compared to unmodified foam. However, the thermal conductivity of all tested foam materials was lower than 25.82 mW/m∙K. The applied modifiers effectively reduced the flammability of rigid polyurethane foams, among others, increasing the oxygen index above 21.4 vol.%, reducing the total heat released by about 41–51% and the rate of heat release by about 2–52%. A correlation between the limiting oxygen index values and both total heat released parameters from the pyrolysis combustion flow calorimetry and cone calorimetry was observed. The correlation was also visible between the value of the heat release capacity (HRC) parameter obtained from the pyrolysis combustion flow calorimetry and the maximum average rate of heat emission (MARHE) from the cone calorimeter test.

## 1. Introduction

Polyurethane foams (PURFs) are the most frequently produced polyurethane materials [1]. The market for flexible and rigid foams accounts for approximately 67% of the global polyurethane market [2]. Closed-cell rigid PURFs are the materials with the lowest thermal conductivity coefficient, which are produced on an industrial scale. For this reason, rigid PURFs are used as thermal insulation of buildings and devices, e.g., refrigerators and freezers [3,4]. Moreover, PURFs have high mechanical strength at a low apparent density (usually 30–70 kg/m^3^), which makes them suitable for use as cores of sandwich panels [5,6,7]. However, unmodified PURFs are flammable, therefore it is important to reduce their flammability using for example flame retardants in their production [8].

There are two types of flame retardants—additive and reactive—mostly used in the production of polymer materials. Additive flame retardants are not chemically bound to the polymer matrix, so they can migrate from the material. Moreover, they often have poor compatibility with the polymers and may reduce their mechanical properties. However, due to their simpler structure, they are cheaper than reactive flame retardants [5,9,10,11]. The advantages of additive flame retardants are their ease-of-use and effective action [12]. Reactive flame retardants have functional groups in their structure, so they can be covalently bonded to the polymer chain. The incorporation of a modifier in the foam structure allows for a permanent reduction of flammability [13]. Reactive flame retardants may affect the production of PURFs, changing their physical and mechanical properties [14]. Currently, the most commonly used reactive flame retardants are derivatives of phosphoric acid [15].

Organic compounds containing chlorine or bromine are popular flame retardants. Even though they effectively reduce the flammability of polyurethane materials, their use is limited, and in some cases even banned. It has been shown that halogen flame retardants emit large amounts of smoke and toxic combustion products during a fire and are harmful to people and the environment [16,17,18]. Therefore, it is important to look for solutions as effective as halogen flame retardants, but safer [12].

Reactive flame retardants can be in the form of oligomeric compounds containing phosphorus, nitrogen, and boron atoms [10,13,19,20,21]. Polyols used as reactive flame retardants can be obtained by modifying vegetable oils with substances containing phosphorus atoms using transesterification as well as epoxidation and opening of oxirane rings [5,22,23,24,25]. Low molecular weight reactive flame retardants containing phosphorus, nitrogen, and boron are also used [8,26,27,28,29,30].

Currently, the polyurethane market is increasingly interested in the materials obtained using plant-derived bio-polyols. This is due to the intention to reduce the consumption of petrochemical raw materials in favour of renewable ones [31,32]. The use of bio-polyols in the manufacture of polyurethanes makes their production more environmentally friendly and consistent with the sustainable development policy [33]. Bio-polyols can be obtained from various types of vegetable oils such as rapeseed, sunflower, soybean, palm, castor, and mustard oils [34,35]. The advantage of using vegetable oils is their easy availability and low price [36]. However, most vegetable oils do not have hydroxyl groups in their structure capable of reacting with isocyanate groups. The introduction of hydroxyl groups is most often achieved by epoxidation of double bonds in unsaturated vegetable oil, followed by the opening of oxirane rings [31,37,38], and also by transesterification [39,40] and transamidization [41,42,43].

The aim of this work was to obtain thermal insulating rigid polyurethane foams with a closed-cell structure and reduced flammability using raw materials of plant origin and to analyse the useful properties of foam materials. Therefore, the foam systems were analysed for the foaming process as well as for the characteristic processing times. The thermal insulation properties and other selected physical and mechanical properties as well as the flammability of the obtained foams were also examined. For the production of PURFs, a new bio-polyol 1.6Hex, obtained from rapeseed oil by epoxidation of the double bond and then opening of the oxirane ring using 1,6-hexanediol, was used [44]. Diethyl bis-(2-hydroxyethyl)aminomethylphosphonate (RE) as well as diethyl (hydroxymethyl)phosphonate (RM) were used as reactive flame retardants and dimethyl propane phosphonate (AD) was used as additive flame retardant. The influence of the used mixture of flame retardants on the thermal insulation properties, flammability and other properties of rigid polyurethane foams has not been described in the literature. The problem of reducing the flammability of rigid PURFs has been discussed comprehensively. The influence of the tested flame retardants was analysed not only on the selected parameters of foams flammability, but also on the course of the foaming process of the modified systems, as well as on the cell structure, thermal insulation, and mechanical properties of the obtained foams.

## 2. Result and Discussion

### 2.1. Analysis of the Foaming Process and Processing Times

**Figure 1:**
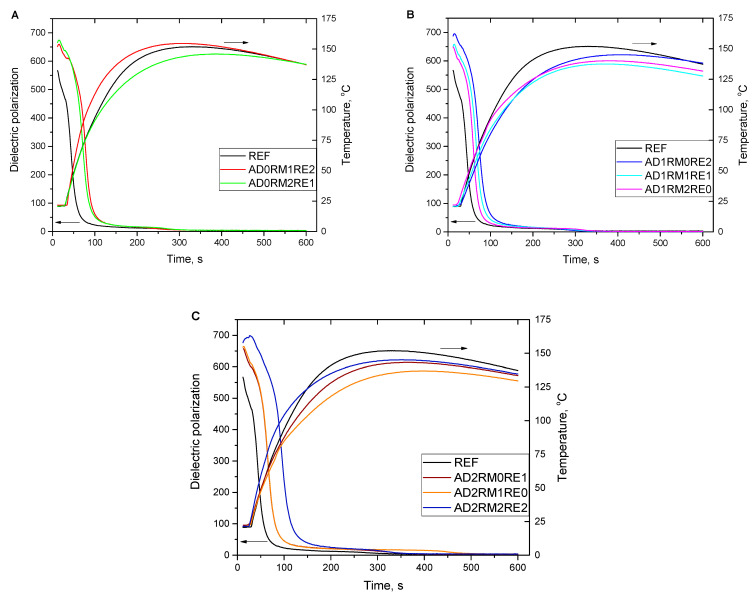
Change of the dielectric polarization and the temperature of the foam core during the foaming process of the tested polyurethane systems.

The analysis of the foaming process was performed using the Foamat^®^ device. The change of dielectric polarization during foaming, which shows the reactivity of the polyurethane system and the temperature in the foam core, were determined [45]. Initial higher values of dielectric polarization of the modified systems at the beginning of the foaming process compared to the reference foam were the result of the presence of hydroxyl groups in the used low-molecular flame retardants. The dielectric polarization curves of the modified systems have a similar slope but are shifted towards longer times (Figure 1). The increase in the content of reactive flame retardants causes a greater shift of the curves, which may indicate higher mobility of flame retardant molecules compared to the petrochemical polyol RF-551.

The maximum temperature of the core of the AD0RM1RE2 foam (containing 10 wt.% of RM and 20 wt.% of RE) was higher than that of the unmodified foam (Figure 1A), and in connection with the course of the curve, it can be concluded that the composition has a higher reactivity compared to the reference foam. In the case of the AD0RM2RE1 foam, the maximum temperature during foaming was reduced from 152 °C 145 °C to for the reference foam, which could be due to a reduction in the reactivity of the composition. For all foams containing 10 wt.% of AD, the temperature in the modified foam core was lower than for the reference foam (Figure 1B). The highest core temperature among foams with 10 wt.% of AD had the AD1RM0RE2 foam, equal to 145 °C, and the lowest temperature, equal to 137 °C, had the AD1RM2RE0 foam. In the case of foams with the addition of 20% AD (Figure 1C), the maximum temperature in the foam core was also lower than for the unmodified foam. The lowest temperature was characteristic for the AD2RM1RE0 foam, equal to 137 °C, and the highest AD2RM2RE2, equal to 145 °C. As a result, it can be concluded that RE is more reactive than RM. The higher reactivity of the composition with the addition of 20 wt.% of RE flame retardant was due to its higher hydroxyl number compared to RM. Therefore, in compositions with a higher content of the reactive flame retardant RE compared to RM, there is a higher content of hydroxyl groups that can generate a greater amount of heat in an exothermic reaction with isocyanate groups.

**Figure 2:**
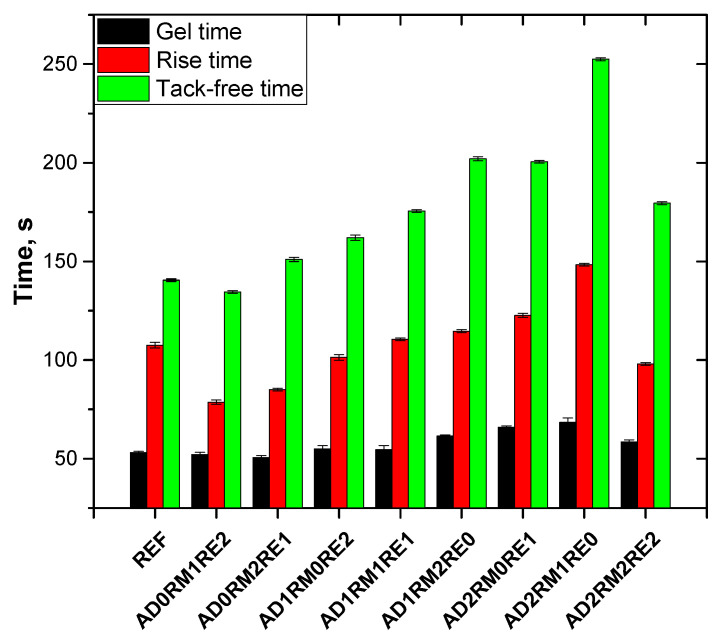
Characteristic processing times of the tested polyurethane foam compositions.

During the manufacture of PURFs, characteristic processing times such as gel time, rise time, and tack-free time were determined (Figure 2), which determine the reactivity of the composition [15]. The gel time of the systems without AD was similar to the reference foam, but the growth time was shortened by 27% and 21% and the dry face time was shortened by 4% and increased by 7% for the AD0RM1RE2 and AD0RM2RE1 foam, respectively, compared to the reference foam. For foams with the addition of 10 wt.% of AD, the shortest processing times were observed for AD1RM0RE2 and the longest for AD1RM2RE0, however, they were longer than for foams that did not contain AD. The same relationship was observed for the AD2RM0RE1 and AD2RM1RE0 foams. In this case, the processing times also increased compared to the composition with the addition of 10 wt.% of AD. This fact shows that RE is more reactive than RM and that AD reduces the reactivity of the composition, thereby increasing the processing times. The extension of the processing times of the compositions with the increase in the addition of AD may result from the reduction of the content of hydroxyl groups per unit weight of the composition. The greater reactivity of RE may be the result of a higher hydroxyl number compared to RM. A higher hydroxyl number of a substance means that in a mass unit there are more hydroxyl groups, so more heat is released during the reaction in a given mass.

### 2.2. Analysis of the Cellular Structure

The cellular structure of PURFs significantly affects their mechanical and thermal insulation properties [46]. The microphotographs of the cellular structure of the tested foams were taken in both cross-sections parallel and perpendicular to the foam rise direction due to the anisotropic structure of the obtained foams. The modified foams in each case had a lower number of cells per 1 mm^2^, and a higher average cell surface in the cross-section parallel to the foam rise direction (Table 1) compared to the reference foam. The modified foams also had a slightly higher anisotropy index compared to the unmodified foam, except AD2RM2RE2 foam. The lower anisotropy of the foams with the highest content of flame retardants is a result of shrinkage and a cell structure distribution in the foam materials. Among foams without the addition of AD but modified with reactive flame retardants, the AD0RM2RE1 foam had the highest number of cells per 1 mm^2^, the highest average cell density, and the lowest average cell surface. This material also had a higher anisotropy index in the cross-section parallel to the rise direction compared to the reference material, which may be due to the highest foam growth rate of the resulting foam materials. In the case of foams containing 10 wt.% of AD, the anisotropy index was close to 1.17. The foam materials AD1RM0RE2 and AD1RM2RE0 had comparable parameters of the cell structure. The material D20V20R0 was characterized by the highest number of cells and the smallest cell surface of the foams with the addition of 20 wt.% of AD. A difference in the anisotropy index of the AD2RM0RE1 and AD2RM1RE0 foams are within the measurement error.

The modified foams also had a lower number of cells and a higher average cell surface in the cross-section perpendicular to the foam rise direction compared to the unmodified foam (Table 2). The anisotropy index of the modified foams was slightly lower than for the reference foam. Among the foams modified with the flame retardants, the foam material AD1RM0RE2 was characterized by the highest number of cells per 1 mm^2^ in the cross-section perpendicular to the foam rise direction. The foams without AD had a higher average cell surface area in a cross-section perpendicular to the foam rise direction compared to the other obtained PURFs.

**Figure 3:**
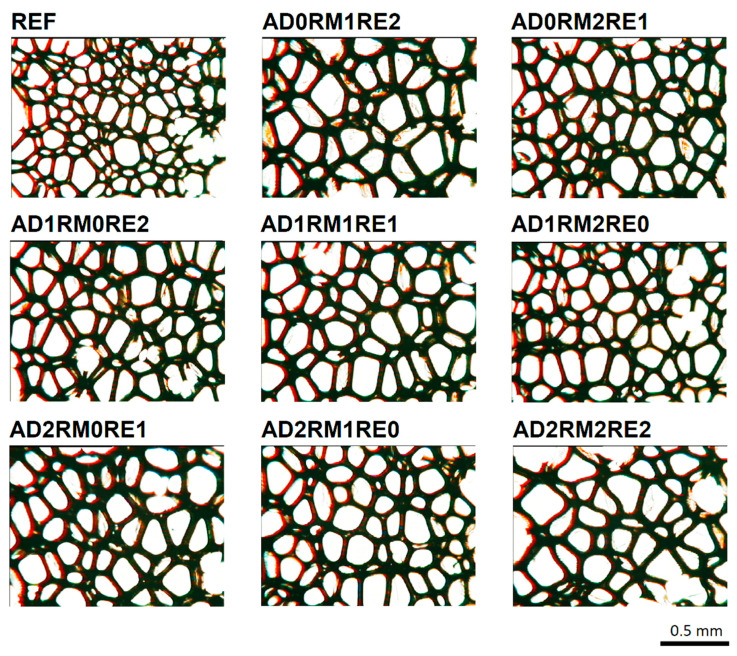
Microphotographs of the foam cell structure in the cross-section parallel to the foam rise direction.

On the microphotographs of the cell structure in the cross-section parallel to the foam rise direction (Figure 3), it can be seen that the modified foams have fewer but larger cells and thicker cell walls compared to the reference material. The cells are characteristically elongated in the direction of the foam growth due to the free growth of the foam material. The AD2RM2RE2 foam material has shrunk, and the cellular structure of the foam has been deformed. Increasing the cell surface area and decreasing the cellular density of modified foams may result from the lower reactivity of the system and the extension of the processing times in relation to the unmodified foam [47].

**Figure 4:**
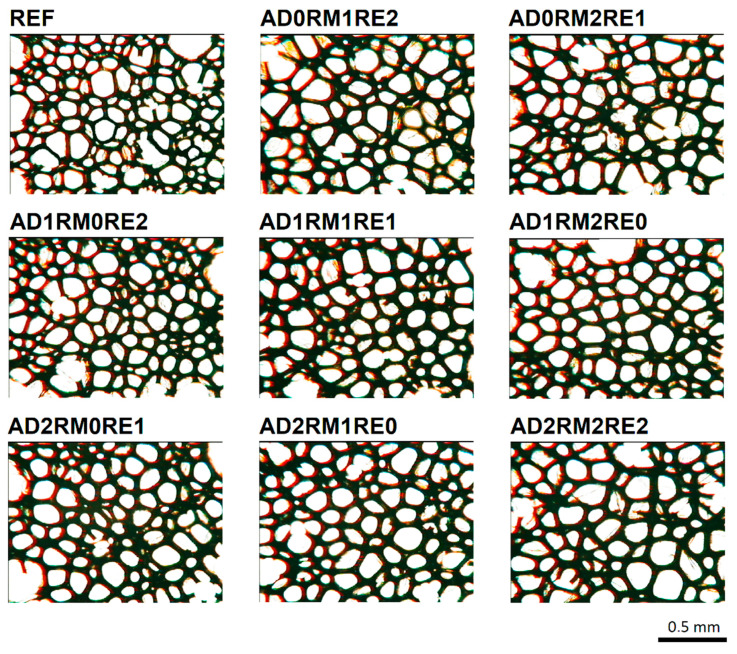
Microphotographs of the foam cell structure in the cross-section perpendicular to the foam rise direction.

The microphotographs of the cell structure in the cross-section perpendicular to the foam rise direction (Figure 4) similarly show that the cell walls of the modified foams are thicker than in the case of the reference foam. The shape of the cells is not elongated in one direction as a result of the rise direction of the foam material. However, the anisotropy index in the cross-section perpendicular to the foam rise is not exactly 1, which may be due to the asymmetrical foam rise and a differential interaction of rising foams within the mould walls.

The closed-cell content of the obtained PURFs is shown in Table 3. This significantly influences the thermal conductivity coefficient of rigid PURFs [48]. For the modified foams, the content of closed cells decreased below 90%. In the case of the foams without the addition of AD, the AD0RM1RE2 foam was characterized by the highest content of closed cells. The foams containing 10 wt.% of AD had a similar closed-cell content of about 86%. The foam materials with the addition of 20 wt.% of AD contained approximately 89% closed cells, except the AD2RM2RE2 foam, which may be due to the deformation of its cell structure. A difference in the content of closed cells for the foams containing 10% or 20 wt.% of AD was within the measurement error. However, when a larger amount of reactive flame retardants was used, this parameter was slightly decreased. A high closed-cell content (>85%) is suitable for foam materials with good thermal insulation properties. The high content of closed cells allows the used blowing agent to be retained in the foam pores, and as a result, a material with a low thermal conductivity coefficient can be obtained [49].

### 2.3. Analysis of Apparent Density and Thermal Conductivity

The apparent density of the reference foam was 36.8 kg/m^3^, and as the content of AD in the foam material increased, the apparent density increased to about 40 kg/m^3^ for foams containing 20 wt.% of AD (Table 4). The worst thermal insulation properties had foams containing 30% of reactive flame retardants, which may be due to the lowest average cell density, as well as the larger cross-sectional area of cells compared to other foam materials obtained. In the case of foams containing 10 wt.% of AD, the thermal insulation properties deteriorated compared to the reference foam, except the AD1RM2RE0 foam, whose thermal conductivity coefficient was similar to that of the unmodified foam. This value of the thermal conductivity of the foam containing 10 wt.% of AD and 20 wt.% of RM may result from the highest cellular density among modified foams and a higher apparent density, which decreased the share of the radiated heat transfer [50]. The foams with the addition of 20 wt.% of AD had about 2.5% higher thermal conductivity compared to the reference foam. Worse thermal insulation properties of modified foams may result from a lower average cell density, larger cell surface, as well as a lower content of closed cells compared to the reference foam. Increasing the anisotropy coefficient of modified foams in the cross-section parallel to the foam rise direction could also reduce the thermal insulation properties because the thermal conductivity coefficient was tested in the cross-section parallel to the rise direction of the foam material. This is particularly evident for the AD0RM1RE2 and AD0RM2RE1 foams, which had the highest thermal conductivity due to the fact that they had one of the lowest cell densities and the highest anisotropy index among the obtained rigid PURFs.

### 2.4. Analysis of the Mechanical Properties

The compressive strength test was performed parallel and perpendicular to the direction of foam rise due to the anisotropic cell structure the obtained foams. The compression stress and Young’s modulus were determined (Table 5). In the case of foams without the addition of AD, the compressive strength and modulus parallel to the foam rise were similar to the value for the reference foam, but in the perpendicular direction, the compressive strength decreased from 131.3 kPa for the reference foam to 98.2 and 90.3 kPa for AD0RM1RE2 and AD0RM2RE1 foams, respectively. The Young’s modulus in the perpendicular direction also decreased by about 28% compared to the reference foam. For foams containing 10 wt.% of AD, the AD1RM1RE1 foam showed the best mechanical properties in the parallel and perpendicular directions. Only this foam material achieved greater compressive strength parallel to the foam rise than the reference foam, which could be due to the higher apparent density than the reference foam. The values of the maximum stress and the modulus of the foams in the direction parallel to the rise direction of the foam containing 20 wt.% of AD were slightly lower than for the unmodified foam, except for the AD2RM2RE2 foam, which had the worst mechanical properties among all the obtained foam materials. In the perpendicular direction, the AD2RM1RE0 foam had the highest compressive strength and modulus among the obtained materials modified with flame retardants. The mechanical strength could have been influenced by the anisotropy coefficient. It was observed that an increase of the anisotropy coefficient in the cross-section parallel to the direction of the foam rise resulted in a significant reduction of the compressive strength in the perpendicular direction and thus an increase in the compressive strength ratio in the parallel direction to the compressive strength in the perpendicular direction. A similar correlation was also observed by other authors and has been described in the literature [51]. Despite an increase of the anisotropy index parallel to the foam rise direction, the compressive strength in the parallel direction of the modified foams decreased, except for the AD1RM1RE1 foam. This may be due to the lower closed-cell content, the larger cross-sectional area of cells, and the lower average cell density compared to the reference foam.

The compressive strength of rigid PURFs largely depends on the apparent density [52]. For this reason, the obtained results of the maximum compressive stress were normalized to the apparent density of 40 kg/m^3^ according to the Formula (1) [53]:

(1) $$\sigma_{\mathrm{norm}}{=\sigma }_{i}{\left(\frac{40}{\rho_{i}}\right)}^{2.1}\;$$

where: σ_i_ is the compressive strength of the sample in kPa, ρ_i_ is the apparent density of the sample in kg/m^3^ σ_norm_ is the normalized compressive strength of the sample in kPa.

**Figure 5:**
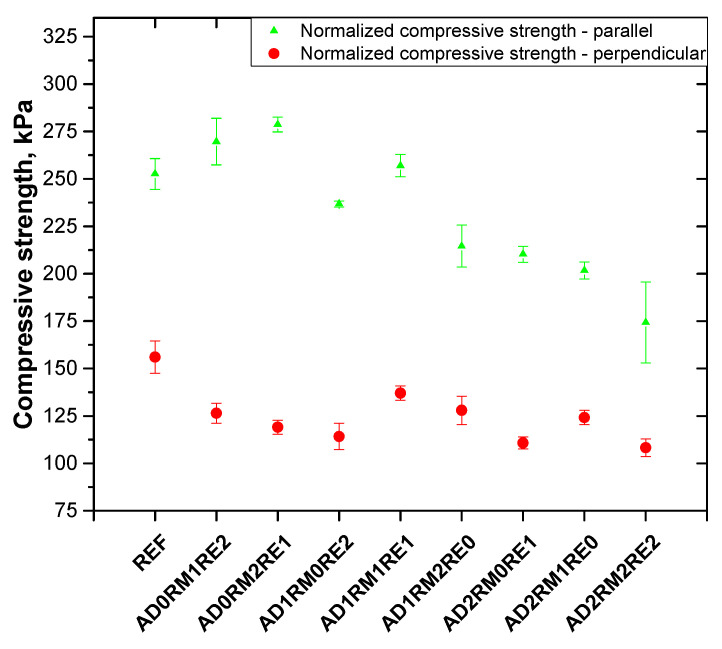
Normalized compressive strength of obtained polyurethane foams.

Due to lower apparent densities, the reference foam and modified foams containing 0% and 10% AD had a higher normalized compressive strength compared to the results not normalized with regard to density (Figure 5). The normalized compression strength in the direction perpendicular to the foam rise is similar for modified foams. It can be seen that the normalized compressive strength is in any case higher than 100 kPa, which will prevent the foam from changing its linear dimensions [54]. It follows that it is more advantageous to obtain rigid PURFs with a density close to 40 kg/m^3^. 

The flame retardant modified PURFs had higher brittleness compared to the reference foam (Table 5). The greatest increase in brittleness was observed for foams without the addition of AD and with the addition of 10 wt.% of AD, which may be due to the lower apparent density of the foams and the higher content of rigid segments, resulting from the incorporation of short-chain flame retardants into the polymer structure.

### 2.5. Analysis of Flammability

**Figure 6:**
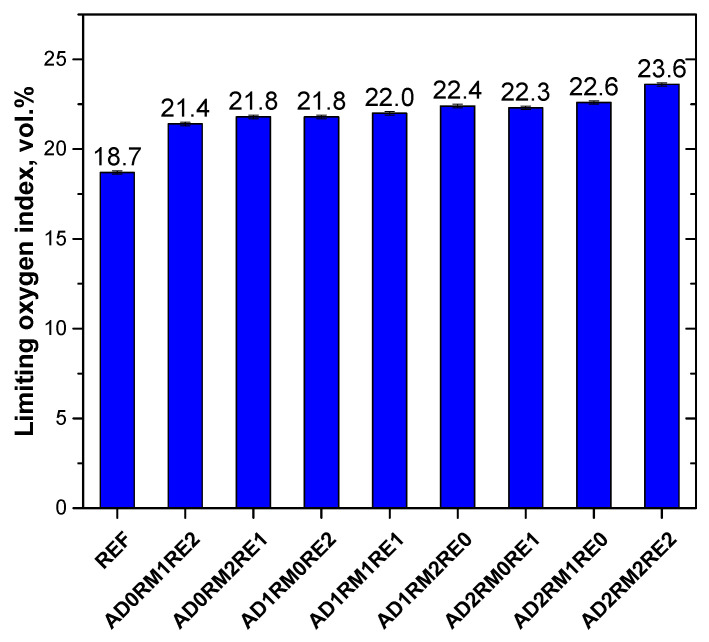
Limiting oxygen index of obtained rigid polyurethane foams.

Limiting oxygen index (LOI) analysis is one of the methods used to determine the flammability of polymers. LOI specifies the minimum oxygen concentration at which the sample will burn [55,56]. All obtained foams with the addition of flame retardants had an oxygen index above 21% (Figure 6). In the case of foams with AD content in the amount of 0, 10, and 20 wt.% in relation to the weight of polyol components, the highest oxygen index was found for the AD0RM2RE1, AD1RM2RE0, and AD2RM2RE2 foams, respectively, and therefore with the highest amount of RM, which may result from a higher phosphorus content in this flame retardant. The increase in AD content also influenced the increase of the limiting oxygen index.

**Figure 7:**
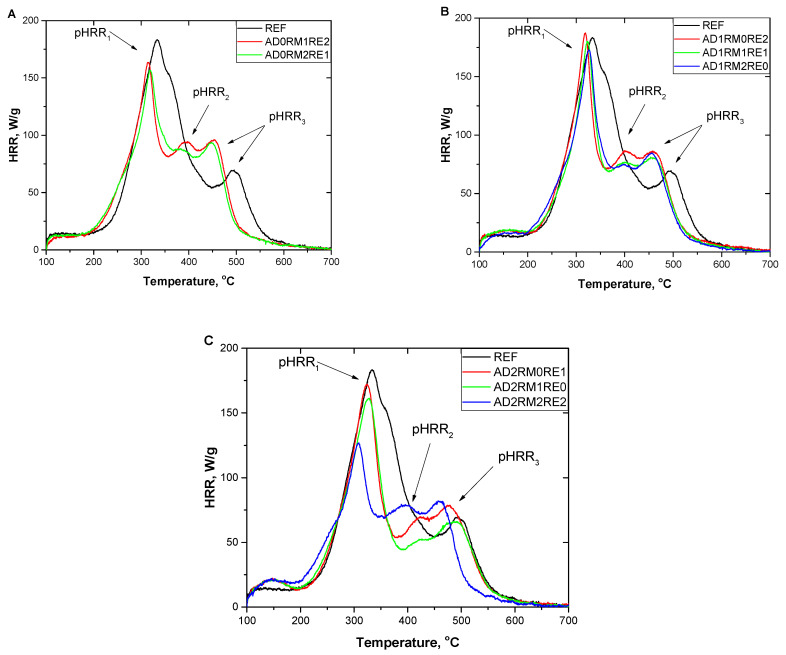
HRR curves of foams without AD (**A**), with 10% AD (**B**), and with 20% AD (**C**).

Combustion parameters were also investigated using a pyrolysis combustion flow calorimeter (PCFC). Parameters such as total heat released (THR), heat release capacity (HRC), the highest heat release rates at individual stages of foam decomposition (pHRR), temperatures at which the heat release rate was the highest, and the heat release rate versus temperature, were determined. Detailed data are presented in Table 6. As can be seen in Figure 7, the thermal decomposition of the reference foams takes place mainly in two stages. The first stage takes place at temperatures ranging from 200 °C to 450 °C, and the second stage at temperatures from 450 °C to 600 °C. On the other hand, modified foams decompose in three stages. The first decomposition stage, which is characterized by the fastest heat release, takes place at temperatures of 200–350 °C, the second and third stage with a similar heat release rate, respectively, at 350–460 °C and 460–600 °C. The appearance of the pHRR_2_ peak in modified foams may result from the separation of the wide pHRR_1_ peak from the reference foam into a narrow pHRR_1_ peak in the modified foams and a low pHRR_2_ peak. The first stage of decomposition of foams modified with more RM in relation to RE begins at lower temperatures, which is related to the lower decomposition temperature of RM (approximately 200 °C) than RE (approximately 225 °C). This fact shows that RM starts its flame retardant action at a lower temperature, limiting the combustion process as soon as it starts, which may prevent the fire from developing.

The addition of flame retardants reduced the total heat released (Table 6). The lowest THR and HRC values with the AD content equal to 0, 10, and 20% were found in the foams containing the highest amount of RM. The combination of AD and RM allowed the most effective reduction of total heat release compared to other compositions. The PURFs containing 10% AD had a similar ability to release heat and pHRR_1_ compared to the reference foam. The AD-free foam materials had an HRC value of about 175 J/g·K. In the case of foams with the addition of 20% AD, the highest HRC value was found for the AD2RM0RE1 material, and the lowest AD2RM2RE2, equal to 189 J/g·K and 136 J/g·K, respectively, so the increase the content of RM in these foams resulted in a reduction of the heat release capacity as well as pHRR_1_. The temperature at which pHRR_1_ is reached has decreased after the addition of flame retardants. For AD-free foams, this temperature decreased by about 16 °C. In the case of materials with 10% AD content, there was a decrease in Temp_1_ for the AD1RM0RE2 foam by 14 °C, and as the RE content decreased, this difference decreased to 9 °C for the AD1RM2RE0 foam compared to the reference foam. Materials containing 10 wt.% of reactive flame retardants achieved pHRR1 at a temperature about 7 °C lower than the unmodified foam. However, the greatest reduction in Temp_1_ was observed for the AD2RM2RE2 material.

Only visible in modified foams, pHRR_2_ for constant AD contents of 0, 10, and 20 wt.% decreases with the increasing RM content. The addition of modifiers increased the pHRR_3_, except for the AD2RM1RE0 foam. Increasing the content of RM and decreasing the content of RE resulted in a decrease in pHRR3. The pHRR_2_ peak reaches its maximum at the lowest temperature of 392 °C for the AD0RM1RE2 foam and the highest temperature of 426 °C for AD2RM0RE1 and AD2RM1RE0. The peak of the last stage of modified foams decomposition is achieved at a lower temperature than for the reference foam. The lowest pHRR_3_ temperature had foams with a total level of reactive flame retardants of 30 wt.%, and the highest temperature for materials containing 10 wt.% of reactive flame retardants.

The analysis with the use of a cone calorimeter allows to determine the behaviour of the material and the time of the combustion process on a laboratory scale and to predict the development of a fire on a large scale [27,57]. Table 7 and Table 8 show the detailed data obtained after the analysis of rigid PURFs using a cone calorimeter, such as ignition time (TTI), total heat released (THR), peak of heat release rate (pHRR), average heat release rate (Av-HRR), maximum average rate of heat emission (MARHE), average effective heat of combustion (Av-EHC), total amount of smoke released per unit area of the material (TSR), total amount of smoke production (TSP), average CO emission (Av-CO), average emission CO_2_ (Av-CO_2_), and the residue after combustion.

In the case of AD-free foams, the lowest value of the THR had the AD0RM2RE1 foam (5.4 MJ/m^2^), which is a 47% reduction in THR compared to the reference foam. The AD0RM2RE1 foam also has a lower Av-EHC than the unmodified foam and pHRR equal to 128.3 kW/m^2^, which is the lowest value among all the obtained materials. The Av-HRR and the MARHE value of the AD0RM1RE2 and AD0RM2RE1 foams were close to each other and lower than in the case of the reference foam by approximately 56% and 60%, respectively. For foams containing 10% AD, the THR, MARHE, and Av-EHC value was the lowest for the AD1RM1RE1 foam, which proves the enhanced flame-retardant effect of PURFs in the case of a combination of AD, RE, and RM. In the case of foam materials containing 20% AD, the THR and Av-HRR values were similar for the AD2RM0RE1 and AD2RM1RE0 foams, equal to 5.6 MJ/m^2^ and 14.9 kW/m^2^, respectively. However, other parameters such as pHRR, MARHE, and Av-EHC were lower for the AD2RM1RE0 foam. The AD2RM2RE2 foam containing significantly more flame retardants compared to other modified foam materials did not have better combustion process parameters, except for MAHRE. It follows that this amount of flame retardants is not optimal in the tested polyurethane system.

Determination of the parameters of smoke emission and gaseous combustion products is very important because they constitute a secondary fire hazard, which is as dangerous to life as in the case of a flame [58]. For foams without AD, the TSR and TSP values decreased significantly, which is characteristic of flame retardants operating in the condensed phase (Table 8) [59,60]. The CO/CO_2_ ratio and the residue after combustion of the AD0RM1RE2 and AD0RM2RE1 foams slightly increased compared to the reference foam. In the case of foams containing 10 wt.% of AD, the lowest TSR and TSP value and the highest residue after combustion had the AD1RM1RE1 foam, which shows that a better effect of reducing smoke emission was achieved when reactive flame retardants were combined. The introduction of AD to the system resulted in an increase in CO release and a reduction in CO_2_, which resulted in a significant increase in the CO/CO_2_ ratio compared to the reference foam. A similar effect was also observed in previous studies [61]. For foams containing 20 wt.% of AD, the TSR, TSP values, as well as the CO/CO_2_ ratio were significantly higher than for unmodified foams and foams containing 10 wt.% of AD. The AD2RM2RE2 foam is characterized by lower TSR and TSP values due to the high content of reactive flame retardants forming a protective charcoal layer on the foam surface.

The increase of the AD content increases the emission of smoke, as well as the CO/CO_2_ ratio compared to unmodified foam. This is the result of the action of AD in the gas phase inhibiting the radical reactions, resulting in more incompletely burned fragments in the foam [61,62].

**Figure 8:**
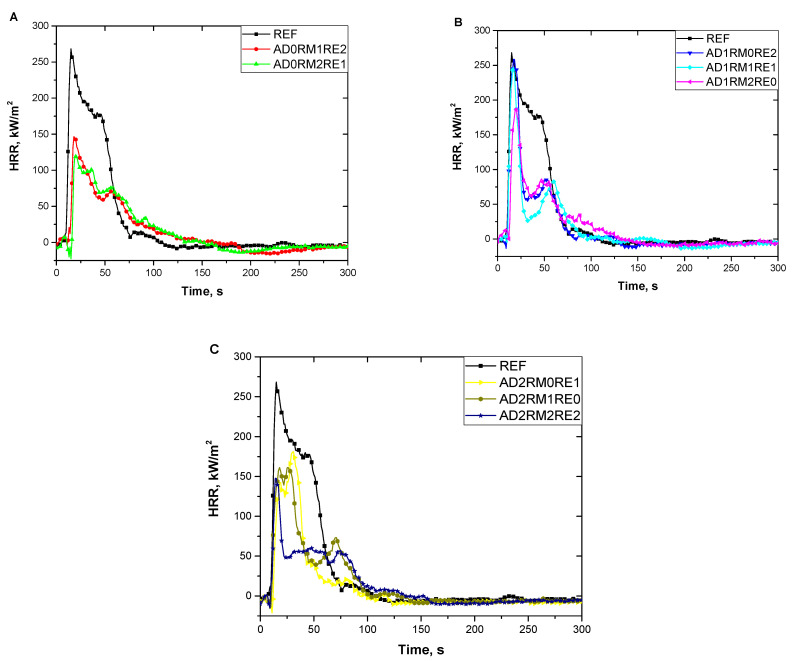
HRR curves of the obtained polyurethane foams without the addition of AD (**A**), containing 10% AD (**B**), containing 20% AD (**C**) from cone calorimetry.

As can be seen in Figure 8, the HRR increases rapidly, reaching pHRR, in which a protective char layer is formed to protect the foam material. After reaching the pHRR, the HRR immediately decreases, and then the second HRR peak is reached, in which the protective layer is destroyed [63]. In Figure 8B, it can be observed that the pHRR value of modified foams is higher than that of other foam materials, but after reaching the peak it decreases the fastest, therefore other parameters such as THR, Av-HRR, and Av-EHC were lower than for other materials.

**Figure 9:**
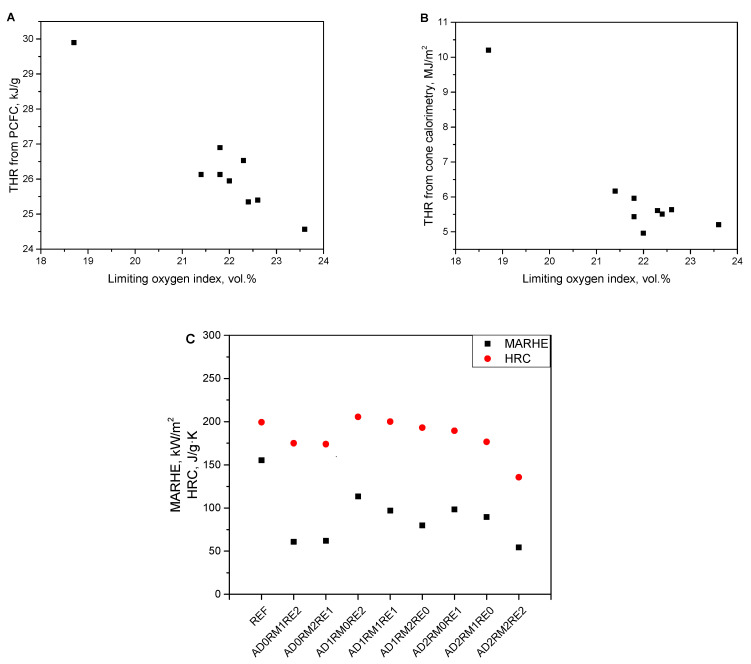
Correlation between the limiting oxygen index and THR from PCFC (**A**), limiting oxygen index and THR from cone calorimetry (**B**) as well as MARHR and HRC (**C**).

A correlation was observed between the LOI and the THR value from the pyrolysis combustion flow calorimetry. When the values of the LOI of the obtained PURFs increase, the TTR (PCFC test) decreases (Figure 9A). Such a relationship was also observed in another study [64]. There is also a relationship between the LOI and THR from cone calorimetry. For the foams obtained, when the value of the LOI increases, the THR from the cone calorimeter test decreases (Figure 9B). The correlation is also visible between the HRC parameter obtained from the PCFC test, which determines the material’s ability of heat emission, and the MARHE parameter, which determines the ability to spread fire [65,66]. For foam materials, due to the decreasing HRC, the MARHE value also decreased (Figure 9C).

## 3. Materials and Methods

### 3.1. Materials 

**Figure 10:**
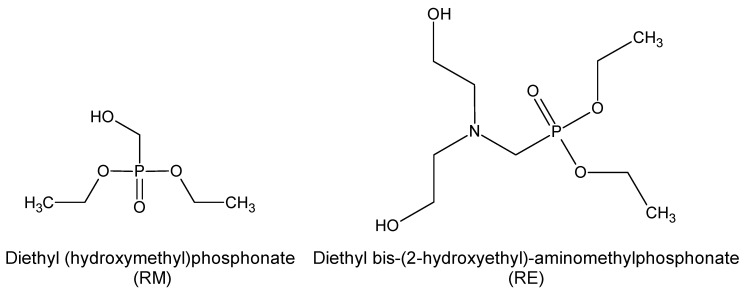
Chemical structure of the reactive flame retardants RM and RE.

The following raw materials were used for the manufacture of PURFs: Rokopol^®^ RF-551 with a hydroxyl number of 420 mgKOH/g and viscosity of 4000 mPa·s provided by PCC Rokita S.A. (Brzeg Dolny, Poland) as a petrochemical polyol; bio-polyol 1.6Hex with a hydroxyl number of 217 mgKOH/g, water content of 0.25 wt.%, and viscosity of 2050 mPa·s. The 1.6Hex bio-polyol was synthesized in the Department of Chemistry and Technology of Polymers at the Cracow University of Technology in Poland by epoxidation of rapeseed oil and opening oxirane rings with 1.6-hexadnodiol [44]. EKOPUR B—polymeric methylene diphenyldiisocyanate (PMDI) with an isocyanate group content of 31 wt.% supplied by Minova Ekochem S.A. (Siemianowice Śląskie, Poland). Polycat^®^ 218, reactive amine catalyst, was supplied by Evonik Industries AG (Essen, Germany). Niax^®^ Silicone L-6915 produced by Momentive Performance Materials Inc. (Waterford, NY, USA) was used as a surfactant. Distilled water was applied as a chemical blowing agent. Dimethyl propane phosphonate (AD) with a phosphorus content of 20.3 wt.% and viscosity of 2.5 mPa·s was provided by Lanxess (Cologne, Germany) as an additive flame retardant. Diethyl (hydroxymethyl) phosphonate (RM) with a hydroxyl number 267 mgKOH/g, phosphorus content of 18.4 wt.% and viscosity of 20 mPa·s supplied by ICL Industrial Products (Amsterdam, The Netherlands) and diethyl bis-(2-hydroxyethyl)-aminomethylphosphonate (RE) with a hydroxyl number 435 mgKOH/g, phosphorus content of 12.2 wt.%, nitrogen content of 5.5 wt.%, and viscosity of 200 mPa·s supplied by PCC Rokita S.A. (Brzeg Dolny, Poland) were used as reactive flame retardants (Figure 10).

### 3.2. Manufacture of the Polyurethane Foams

Rigid PURFs were obtained by the one-step method by vigorously mixing the polyol premix with the isocyanate component, and then pouring the reaction mixture into a mould, allowing the foam to free rise in a vertical direction. The polyol premix consisted of petrochemical polyol, bio-polyol 1.6Hex obtained from rapeseed oil, catalyst, surfactant, and blowing agent. Modified foams were obtained by adding an appropriate amount of reactive and additive flame retardants to the polyol premix. The reactive flame retardants replaced the appropriate amount of RF 551 petrochemical polyol in the polyurethane system. Additive flame retardant (AD) was added in an amount of 0−20% in the relation to the weight of the polyol components. The obtained PURFs were seasoned for 24 h at room temperature. The formulas of applied polyurethane systems are presented in Table 9.

### 3.3. Characterization of Rigid Polyurethane Foams 

The foaming process analysis was performed by FOAMAT^®^ device (Format-Messtechnik GmbH, Freiburg im Breisgau, Germany). During foaming, the temperature in the core of foams and the dielectric polarization were determined. The change in temperature and dielectric polarization shows the reactivity of the polyurethane system. During the manufacture of foams, characteristic processing times were also determined, such as gel time, rise time, and tack-free time using an electronic stopwatch. 

The measurement of the thermal conductivity coefficient was carried out with a Laser Comp Heat Flow Instrument Fox 200 (New Castle, DE, USA) according to ISO 8301. The foams were tested 24 h after manufacture. In the apparatus, there was one-way steady heat flow between a heat plate at a temperature of 20 °C and a cold plate at a temperature of 0 °C. The tested samples had dimensions of 200 × 200 × 50 mm^3^.

The apparent density was tested as a ratio of mass and volume of foam materials according to ISO 845. The percentage of closed cells was determined according to ISO 4590. 

The cellular structure of the PURFs was determined on the basis of photos taken with an optical microscope equipped with a camera. The number of cells per 1 mm^2^, average cell density, the average cross-sectional area of cells, and the anisotropy coefficient calculated from the ratio of the height and width of the cells were determined. Average cell density was determined according to the Equation (2) [67]:

(2) $$\mathrm{CD}=N_{\mathrm{PAR}}\cdot {\left(N_{\mathrm{PERP}}\right)}^{\frac{1}{2}}$$

where N_PAR_ is the number of cells in the microphotographs in the cross-section parallel to the foam rise and N_PERP_ is the number of cells in the microphotographs in the cross-section perpendicular to the foam rise.

The compressive strength test (according to ISO 844) of rigid PURFs was carried out in the two directions parallel and perpendicular to the direction of foam rise. Cylindrical samples with a diameter and height of 40 mm were used for the test.

The brittleness test of PURFs was carried out in an oak box filled with 24 oak cubes with an edge of 20 mm, which rotated at a speed of 60 rpm for 10 min according to ASTM C-421. The brittleness of the foams was determined as the percentage weight loss of 12 cubes with dimensions of 25 × 25 × 25 mm^3^.

The flammability of the samples was determined by the limiting oxygen index according to ISO 4589–2. The PURFs were tested with a pyrolysis combustion flow calorimeter (PCFC) manufactured by Fire Testing Technology Ltd. (East Grinstead, UK). The test was performed in accordance with ASTM D7309 method A. The pyrolysis tests of the samples under nitrogen atmosphere were carried out at temperatures of 100–750 °C, with a heating rate of 1 °C/s. The weight of the samples was approximately 2 mg. A synthetic air mixture with a volume ratio of N_2_/O_2_ = 80/20 was supplied to the apparatus. The study allowed for the determination of combustion process parameters such as total heat release (THR), heat release capacity (HRC), heat release rate (HRR), and the temperatures at which the local maximum of HRR was reached.

The combustion behaviour of the foams was also determined using FTT cone calorimeter (Fire Testing Technology Ltd., East Grinstead, UK). The samples with dimensions 100 × 100 × 10 mm^3^ were subjected to an external heat source of 35 kW/m^2^ for 300 s according to ISO 5660–1. The following parameters were determined: ignition time (TTI), total heat released (THR), maximum heat release rate (pHRR), average heat release rate (Av-HRR), the maximum average rate of heat emission (MARHE), average effective heat of combustion (Av-EHC), total amount of smoke released per unit area of material (TSR), total smoke production (TSP), average CO emission (Av-CO), average CO_2_ emission (Av-CO_2_), and char residue.

## 4. Conclusions

Closed-cell rigid polyurethane foams containing the additive flame retardant and two reactive flame retardants have been successfully obtained. The addition of modifiers changed the reactivity of the tested polyurethane systems. It caused a decrease in cellular density and an increase the anisotropy coefficient, which reduced the compressive strength, as well as worsened the thermal insulating properties of the obtained polyurethane foams. 

Foams containing 30 wt.% of reactive flame retardants and the AD1RM0RE2 foam, which were the only ones that did not have a higher apparent density than the unmodified foam, had a compressive strength lower than 100 kPa perpendicular to the direction of foam rise, which may cause the foam materials to not be dimensionally stable throughout their service life [1,54]. Therefore, it would be more advantageous to obtain foams with a slightly higher apparent density. In addition, the AD2RM2RE2 foam has shrunk, so it can be concluded that it has been modified with too much of an addition of flame retardants.

The addition of flame retardants effectively increased the limiting oxygen index and lowered the parameters of the combustion process obtained from the pyrolysis combustion flow calorimetry as well as the cone calorimetry. For systems containing 30 wt.% of reactive flame retardants and 0, 10, and 20 wt.% of the additive flame retardant, respectively, the least flammable were the AD0RM2RE1, AD1RM2RE0, and AD2RM1RE0 foams, thus those containing the highest amount of RM due to the higher phosphorus content in this reactive flame retardant. The AD2RM2RE2 foam had only a slightly reduced certain combustion parameters, and it follows that the addition of such large amounts of flame retardants is not economically viable.

The foams containing only reactive flame retardants had lower TSP and TSR values, which proves the effective flame retardant action of these substances in the condensed phase, creating a charred layer limiting the heat flow to the material, as well as flammable gaseous products of decomposition of the material into the flame zone. Increasing the content of the additive flame retardant increases both of these parameters as it mainly works in the gas phase.

## Figures and Tables

**Table 1:** Parameters of the cellular structure of rigid polyurethane foams in the cross-section parallel to the foam rise direction.

Foam Symbol	Number of Cells per 1 mm^2^	Average Cross-Sectional Area of Cell·10^3^, mm^2^	Anisotropy Index
**REF**	53 ± 4	9.27 ± 0.59	1.10 ± 0.04
**AD0RM1RE2**	31 ± 1	13.65 ± 0.69	1.18 ± 0.05
**AD0RM2RE1**	36 ± 2	12.39 ± 0.93	1.21 ± 0.01
**AD1RM0RE2**	39 ± 2	11.73 ± 0.72	1.17 ± 0.06
**AD1RM1RE1**	31 ± 2	14.72 ± 0.81	1.18 ± 0.06
**AD1RM2RE0**	40 ± 2	11.77 ± 0.86	1.16 ± 0.05
**AD2RM0RE1**	32 ± 2	13.15 ± 0.87	1.14 ± 0.04
**AD2RM1RE0**	37 ± 3	11.55 ± 0.86	1.11 ± 0.07
**AD2RM2RE2**	31 ± 2	14.25 ± 0.63	1.05 ± 0.04

**Table 2:** Parameters of the cell structure of rigid polyurethane foams in the cross-section perpendicular to the foam rise direction.

Foam Symbol	Number of Cells per 1 mm^2^	Average Cross-Sectional Area of Cell·10^3^, mm^2^	Anisotropy Index
**REF**	66 ± 3	6.95 ± 0.73	0.94 ± 0.04
**AD0RM1RE2**	48 ± 3	9.76 ± 0.88	0.86 ± 0.01
**AD0RM2RE1**	47 ± 3	9.43 ± 0.28	0.88 ± 0.03
**AD1RM0RE2**	56 ± 3	8.27 ± 0.69	0.91 ± 0.01
**AD1RM1RE1**	51 ± 2	8.30 ± 0.90	0.88 ± 0.04
**AD1RM2RE0**	52 ± 2	8.58 ± 0.40	0.88 ± 0.03
**AD2RM0RE1**	51 ± 3	8.24 ± 0.83	0.91 ± 0.03
**AD2RM1RE0**	50 ± 3	8.27 ± 0.71	0.87 ± 0.03
**AD2RM2RE2**	48 ± 3	8.70 ± 0.77	0.90 ± 0.03

**Table 3:** Closed-cell content of the obtained polyurethane foams and average cell density.

Foam Symbol	Content of Closed Cell, %	CD, Number of Cells/mm^3^
**REF**	90.8 ± 0.7	432 ± 7
**AD0RM1RE2**	88.8 ± 0.6	216 ± 3
**AD0RM2RE1**	86.3 ± 0.7	244 ± 4
**AD1RM0RE2**	86.0 ± 0.7	295 ± 4
**AD1RM1RE1**	86.1 ± 0.4	254 ± 5
**AD1RM2RE0**	86.4 ± 0.6	287 ± 3
**AD2RM0RE1**	88.4 ± 1.3	227 ± 3
**AD2RM1RE0**	89.2 ± 0.8	263 ± 4
**AD2RM2RE2**	87.9 ± 0.9	212 ± 3

**Table 4:** Thermal conductivity coefficient, apparent density, and content of the closed cell of rigid polyurethane foams.

Foam Symbol	Apparent Density, kg/m^3^	Thermal Conductivity Coefficient, mW/m·K	Content of Closed Cell, %
**REF**	36.8 ± 0.4	24.36 ± 0.08	90.8 ± 0.7
**AD0RM1RE2**	35.5 ± 0.4	25.82 ± 0.83	88.8 ± 0.6
**AD0RM2RE1**	35.1 ± 1.0	25.37 ± 0.41	86.3 ± 0.7
**AD1RM0RE2**	36.8 ± 0.5	25.24 ± 0.01	86.0 ± 0.7
**AD1RM1RE1**	37.3 ± 0.8	24.74 ± 0.06	86.1 ± 0.4
**AD1RM2RE0**	38.4 ± 0.1	24.38 ± 0.16	86.4 ± 0.6
**AD2RM0RE1**	39.4 ± 0.1	25.02 ± 0.24	88.4 ± 1.3
**AD2RM1RE0**	40.4 ± 0.5	24.89 ± 0.06	89.2 ± 0.8
**AD2RM2RE2**	39.7 ± 0.1	24.94 ± 0.20	87.9 ± 0.9

**Table 5:** Mechanical properties of obtained rigid polyurethane foams.

Foam Symbol	Parallel		Perpendicular		Brittleness, %
	Compressive Strength, kPa	Modulus, MPa	Compressive Strength, kPa	Modulus, MPa	
**REF**	212.6 ± 6.8	5.17 ± 0.34	131.3 ± 7.2	3.33 ± 0.16	3.04 ± 0.23
**AD0RM1RE2**	209.4 ± 9.6	5.15 ± 0.32	98.2 ± 4.1	2.41 ± 0.05	4.90 ± 0.43
**AD0RM2RE1**	211.2 ± 3.0	4.93 ± 0.17	90.3 ± 2.8	2.41 ± 0.11	5.91 ± 0.31
**AD1RM0RE2**	199.2 ± 1.3	4.91 ± 0.17	96.0 ± 5.9	2.53 ± 0.14	6.06 ± 0.50
**AD1RM1RE1**	221.9 ± 5.1	5.00 ± 0.06	118.3 ± 3.3	2.84 ± 0.17	5.29 ± 0.24
**AD1RM2RE0**	197.0 ± 10.1	4.72 ± 0.12	117.5 ± 6.8	2.74 ± 0.10	4.22 ± 0.33
**AD2RM0RE1**	203.5 ± 4.1	4.81 ± 0.05	107.2 ± 3.1	2.63 ± 0.07	3.35 ± 0.32
**AD2RM1RE0**	205.5 ± 4.6	5.00 ± 0.13	126.4 ± 3.8	3.01 ± 0.05	3.70 ± 0.22
**AD2RM2RE2**	171.7 ± 21.1	4.61 ± 0.64	106.7 ± 4.6	2.25 ± 0.16	3.93 ± 0.44

**Table 6:** Combustion parameters obtained with the pyrolysis and combustion microcalorimeter (PCFC).

Foam Symbol	THR, kJ/g	HRC, J/g·K	Temp_1_, °C	pHRR_1_, W/g	Temp_2_, °C	pHRR_2,_ W/g	Temp_3_, °C	pHRR_3_, W/g
**REF**	29.9 ± 0.3	199 ± 3	334 ± 2	182.0 ± 1.7	-	-	488 ± 7	67.2 ± 6.2
**AD0RM1RE2**	26.2 ± 0.5	175 ± 5	317 ± 2	160.4 ± 4.3	392 ± 3	91.6 ± 5.3	450 ± 2	94.3 ± 3.1
**AD0RM2RE1**	26.1 ± 0.2	174 ± 7	319 ± 1	158.9 ± 6.3	378 ± 3	89.8 ± 3.0	448 ± 1	93.2 ± 1.0
**AD1RM0RE2**	26.9 ± 0.6	206 ± 1	320 ± 1	187.9 ± 1.7	401 ± 1	89.7 ± 3.1	456 ± 2	90.3 ± 3.4
**AD1RM1RE1**	26.0 ± 0.1	200 ± 6	323 ± 1	182.2 ± 3.4	392 ± 5	77.1 ± 0.7	456 ± 2	81.8 ± 1.8
**AD1RM2RE0**	25.4 ± 0.4	193 ± 8	324 ± 1	176.4 ± 5.2	401 ± 3	72.6 ± 3.4	457 ± 5	82.3 ± 2.9
**AD2RM0RE1**	26.5 ± 0.4	189 ± 3	325 ± 2	172.9 ± 1.8	426 ± 2	68.6 ± 1.1	477 ± 2	77.1 ± 2.0
**AD2RM1RE0**	25.4 ± 0.7	177 ± 4	327 ± 3	162.8 ± 2.7	426 ± 1	53.3 ± 1.6	486 ± 2	65.8 ± 1.5
**AD2RM2RE2**	24.6 ± 0.3	136 ± 5	308 ± 1	124.0 ± 4.0	394 ± 1	81.8 ± 3.3	458 ± 2	84.6 ± 2.6

**Table 7:** Parameters of the combustion process after analysis with a cone calorimeter.

Foam Symbol	TTI, s	THR, MJ/m^2^	pHRR, kW/m^2^	Av-HRR, kW/m^2^	MARHE, kW/m^2^	Av-EHC, MJ/kg
**REF**	4 ± 1	10.2 ± 1.1	268.7 ± 10.7	31.3 ± 3.9	156 ± 4	12.8 ± 0.6
**AD0RM1RE2**	3 ± 1	6.2 ± 0.4	155.8 ± 12.9	13.6 ± 4.5	61 ± 9	8.1 ± 0.2
**AD0RM2RE1**	3 ± 1	5.4 ± 0.9	128.3 ± 8.8	14.1 ± 2.9	62 ± 6	7.1 ± 0.7
**AD1RM0RE2**	4 ± 1	6.0 ± 0.3	262.3 ± 4.8	15.4 ± 1.0	113 ± 3	6.2 ± 0.3
**AD1RM1RE1**	4 ± 1	5.0 ± 0.8	237.5 ± 13.4	12.7 ± 3.4	97 ± 5	5.1 ± 0.5
**AD1RM2RE0**	5 ± 1	5.5 ± 0.7	201.2 ± 13.6	14.4 ± 3.1	80 ± 2	7.1 ± 0.1
**AD2RM0RE1**	3 ± 1	5.6 ± 0.6	190.2 ± 8.9	14.8 ± 2.9	98 ± 6	5.9 ± 0.3
**AD2RM1RE0**	3 ± 1	5.6 ± 0.5	162.7 ± 8.0	14.9 ± 1.7	90 ± 5	5.1 ± 0.8
**AD2RM2RE2**	2 ± 1	5.2 ± 0.2	139.6 ± 11.4	13.7 ± 1.1	54 ± 1	5.8 ± 0.4

**Table 8:** Smoke emission parameters of the obtained polyurethane foams.

Foam Symbol	TSR, m^2^/m^2^	TSP, m^2^	Av-COY, kg/kg	Av-CO_2_Y, kg/kg	CO/CO_2_ Weight Ratio	Residue, %
**REF**	501 ± 39	4.43 ± 0.34	0.60 ± 0.08	3.87 ± 0.38	0.155 ± 0.004	30.7 ± 3.2
**AD0RM1RE2**	315 ± 44	2.79 ± 0.39	0.64 ± 0.04	3.70 ± 0.33	0.173 ± 0.012	37.0 ± 4.1
**AD0RM2RE1**	360 ± 36	3.18 ± 0.31	0.68 ± 0.08	3.65 ± 0.42	0.187 ± 0.007	35.3 ± 8.9
**AD1RM0RE2**	519 ± 41	4.59 ± 0.36	0.91 ± 0.06	3.29 ± 0.40	0.278 ± 0.018	26.8 ± 4.2
**AD1RM1RE1**	476 ± 28	4.21 ± 0.24	0.99 ± 0.21	3.48 ± 0.71	0.285 ± 0.016	42.7 ± 0.3
**AD1RM2RE0**	598 ± 31	5.29 ± 0.27	1.11 ± 0.17	3.31 ± 0.38	0.336 ± 0.015	30.5 ± 5.2
**AD2RM0RE1**	818 ± 32	7.23 ± 0.28	1.15 ± 0.08	2.57 ± 0.20	0.448 ± 0.008	19.1 ± 3.9
**AD2RM1RE0**	875 ± 32	8.11 ± 0.67	1.25 ± 0.11	2.75 ± 0.28	0.455 ± 0.007	17.0 ± 6.9
**AD2RM2RE2**	453 ± 24	4.01 ± 0.21	0.98 ± 0.04	3.16 ± 0.32	0.311 ± 0.017	29.9 ± 8.5

**Table 9:** Formulas of rigid polyurethane foams.

Foam Symbol	RF-551, g	1.6Hex, g	Polycat^®^ 218, g	L-6915, g	Water, g	PMDI, g	AD, g	RM, g	RE, g
**REF**	60	40	1.5	1.5	3.23	148.00	0	0	0
**AD0RM1RE2**	30					144.73	0	10	20
**AD0RM2RE1**	30					140.27	0	20	10
**AD1RM0RE2**	40					148.80	10	0	20
**AD1RM1RE1**	40					144.33	10	10	10
**AD1RM2RE0**	40					139.87	10	20	0
**AD2RM0RE1**	50					148.40	20	0	10
**AD2RM1RE0**	50					143.94	20	10	0
**AD2RM2RE2**	20					140.67	20	20	20

## Data Availability

Not applicable.
